# Regarding: ‘High-intensity-focused ultrasound in the treatment of primary prostate cancer: the first UK series’

**DOI:** 10.1038/sj.bjc.6605455

**Published:** 2009-12-08

**Authors:** S Eggener, M Gonzalgo, O Yossepowitch

**Affiliations:** 1 Section of Urology, University of Chicago, Chicago, IL, USA; 2 Department of Urology, Stanford University, Stanford, CA, USA; 3 Department of Urology, Rabin Medical Center, Petah Tikva, Israel

**Sir,**

In countries in which PSA-based screening has been widely adopted, prostate cancer has a high incidence-to-mortality ratio (e.g., 6.5 in the United States) (Jemal *et al*, 2008), and after prostatectomy, up to 50% of screen-detected cancers are pathologically characterised as indolent (Steyerberg *et al*, 2007; Jemal *et al*, 2008). Surgery and radiotherapy have an excellent track record of cancer control but are accompanied by risks of urinary and sexual morbidity (Sanda *et al*, 2008). For these reasons, we are enthusiastic about efforts to identify men who are most likely to benefit from treatment, as well as novel paradigms aimed at reducing treatment-related morbidity without compromising effectiveness (Eggener *et al*, 2007, 2009). The article by Ahmed *et al* (2009) describes a commendable effort to investigate the oncological and functional outcomes after whole-gland high-intensity-focused ultrasound (HIFU) for localised prostate cancer.

Most patients who elect treatment of their prostate cancer expect to achieve all three components of an ideal outcome: long-term durable cure of their cancer, maintenance or improvement of urinary function, and preservation of erectile function. The data presented by Ahmed *et al* raise concerns about the ability of HIFU to satisfactorily and reliably achieve these goals.

First, interpretation of cancer recurrence data is complicated by inadequate patient follow-up, conflicting information, and overstated conclusions. Recurrence after HIFU was defined as a PSA nadir >0.5 ng ml^−1^ or a nadir <0.5 ng ml^−1^ and two consecutive rises. However, the mean follow-up in the cohort was slightly less than 1 year. As PSA measurements were obtained every 3 months and roughly a third of the patients were given hormonal therapy to reduce gland size before HIFU, it is likely that most men have not been followed up long enough to meet the criteria for recurrence. Reporting discrepancies were also identified that warrant further clarification. In the Results section, the authors state that the lowest PSA achieved by a patient was 0.12 ng ml^−1^; in contrast, Table 3 states that 35–40% had a PSA <0.05 ng ml^−1^. In addition, 12 months after treatment, the mean PSA is reported to be 0.65 ng ml^−1^, the maximum PSA 1.02 ng ml^−1^, and 58% of patients had a PSA <0.2 ng ml^−1^. According to our calculations, it is impossible for all three of these statements to occur simultaneously. The authors state that cancer control after HIFU is equivalent to radical prostatectomy, as 60% of patients had a PSA <0.2 ng ml^−1^ 2 years after treatment. This exuberant claim of equivalence seems to be premature and unfounded, as 88 and 72% of patients after prostatectomy have a PSA <0.2 ng ml^−1^ at 5 (Nielsen *et al*, 2008) and 10 (Stephenson *et al*, 2006) years, respectively, after treatment. Finally, as inclusion criteria mandate an estimated prostate volume of less than 40 cm^3^, approximately half of the patients are excluded or require androgen deprivation therapy (Pettus *et al*, 2009), which is associated with a significant number of side effects.

Second, after treatment, 24% of patients were treated for a urinary tract infection and 32% required an intervention for urinary debris or urethral stricture. Given the relatively high rate of strictures, longer-term data are required to assess the impact of HIFU on urinary function.

Third, the investigators performed analyses on the basis of two separate definitions of potency: an erection hard enough for penetration ‘much less than half the time’ or ‘about half the time’. Presumptively, for most men with reliable erections before treatment, attaining a satisfactory erection on 50% or fewer attempts would be disappointing and would be considered as a major adverse impact of treatment. In addition, the authors erroneously state in the Conclusions section that two-thirds of the patients had erections sufficient for intercourse. However, this is based on 12 patients meeting the pre-treatment criteria of erections hard enough ‘about half the time’, with 8 maintaining that loose definition after treatment.

Registry trials of HIFU are ongoing and we anticipate longer-term outcome data. On the basis of the early data from this report, we are sceptical whether HIFU is capable of adequately providing the oncological and functional outcomes that patients and physicians are striving to achieve.
